# How much time is enough? Establishing an optimal duration of recording for ambulatory video EEG

**DOI:** 10.1002/epi4.12517

**Published:** 2021-07-16

**Authors:** Hans Klein, Trudy Pang, Jeremy Slater, Richard Eugene Ramsay

**Affiliations:** ^1^ Stratus Irving TX USA; ^2^ Beth Israel Deaconess Medical Center Department of Neurology Harvard Medical School Boston MA USA; ^3^ Department of Neurology University of Texas McGovern Medical School Houston TX USA; ^4^ Neurotech Associates Inc Miami FL USA

**Keywords:** epilepsy, epilepsy monitoring, recording duration, remote care, video electroencephalography

## Abstract

**Objective:**

Ambulatory video EEG allows for extended recording of EEG in the comfort of a patient's home. However, the optimal duration of recording to capture clinical events is yet to be established. The current study uses retrospective analyses to identify an optimal recording duration for at‐home video EEG.

**Methods:**

A retrospective review was performed utilizing an anonymized database of ambulatory video EEG recordings performed between March and September 2020 with a national in‐home EEG provider. Only completed assessments with neurologists' reads of raw data were reviewed, resulting in 3644 unique studies divided into three age cohorts: pediatrics (n = 941), adult (n = 2020), and geriatric (n = 683). Cohorts were characterized by assessment yield and time to first typical clinical event, as well as subsequent typical events over duration of recording.

**Results:**

Frequency distributions reveal over half of first events are captured within 12 hours, but longer recording durations capture a much wider majority of both first typical events, as well as the mean number of subsequent events (5 clinical events). In 72 hours, over 97% of first events were observed in adult and geriatric patients, as well as over 95% of the mean number of subsequent events. In children, time to first event was significantly earlier than either adult or geriatric samples, with 98% of first events, and 92.8% of the mean number of subsequent events being observed in 48 hours.

**Significance:**

These results from a large‐scale, national dataset of patients using in‐home EEG monitoring suggests recording at least 48 hours in duration for children, and at least 72 hours in duration for adult and geriatric samples, is optimal to maximize the likelihood of observing typical clinical events to facilitate diagnosis.

**Key Points**

- Optimal recording duration for at‐home video EEG was examined within pediatric, adult, and geriatric patients.
- Children experience first typical events earlier than adult or geriatric populations.
- For all three groups, extended recordings were significantly related to increased odds of capturing clinical events.
- Ideal duration to optimize capture of clinical events in children is at least 48 hours.
- For adult and geriatric patients, optimal EEG recording is between 48 and 72 hours, with individual patients benefiting from longer assessments.

## 1 INTRODUCTION

The gold standard of epilepsy diagnosis is simultaneous EEG recording with time‐locked video monitoring of clinical events. Extending monitoring provides a greater diagnostic yield over 20–30‐minute routine studies^1^ and increases the likelihood of detecting interictal epileptiform abnormalities such as significant spikes or sharp waves.^2^ However, most prolonged video EEG monitoring studies are ordered in the hopes of recording at least one of the patient's typical ictal events. A long‐standing issue between epileptologists is determining the optimal duration of EEG recording to capture one of these clinical events.

Much of the literature examining optimal recording duration comes from inpatient epilepsy monitoring units (EMUs) and suggests that capture of first event occurs between 2 and 3 days (48 and 72 hours) of recording.^3^, ^4^, ^5^ However, it is difficult to generalize results from EMU research to in‐home EEG monitoring, as routine medication washouts and activation procedures can be achieved with continuous clinical monitoring to increase the likelihood of events occurring sooner.^6^ Such procedures^6^ may not only be unsafe in the outpatient setting, but also uncomfortable or unnecessary for some patients. Although inpatient video EEG monitoring is advantageous for certain diagnostic outcomes,^7^ in‐home video EEG monitoring has demonstrated equitable data quality and diagnostic yield.^8^ Further, the natural environmental stressors of the patient's home may spur certain events that may not otherwise occur in an EMU setting.^7^, ^9^ Therefore, in‐home vide EEG, when used effectively, offers a unique opportunity to reduce some of the burdens associated with costs and scheduling of inpatient assessments^10^ and can support initiatives to improve access to diagnostic testing, specifically for the many epilepsy patients who live in rural regions or underserved communities.^11^ With more flexibility in prescribing prolonged in‐home EEG assessments, determining an optimal duration to observe typical, clinically relevant events is essential.

For in‐home assessments, researchers assessing the diagnostic utility of prolonged EEG in outpatient settings have indirectly examined optimal recording duration. Fox et. al^12^ notes in a small sample of patients with previously nondiagnostic EMU stays (n = 62), half of the sample had sufficient diagnostic information by the end of 72 hours of ambulatory EEG, with 32.3% reaching a diagnosis within 24 hours, and the remaining equally distributed over the following two 24‐hour blocks. Primiani et. al^13^ indicates the average time to first event occurring around 21.8 hours, ranging from 1 to 115 hours after starting the ambulatory recording. Critically, this study's sample included a broad age range (12–101 years), which may have impacted these findings, as optimal duration for event capture may not be equivalent among adult, pediatric,^14^ and geriatric populations^15^ due to differences in the common types of epileptic syndromes in these life stages.

The current study seeks to examine the optimal recording duration for at‐home video EEG by examining the time to first clinically significant event in a large multistate dataset. Using a retrospective review of data from over 3500 outpatient recordings, the timing of typical events are characterized separately within pediatric, adult, and geriatric populations and compared among cohorts to aid in determining optimal durations of recordings for each age cohort.

## 2 METHODS

### 2.1 Study design

This study utilized a retrospective review of anonymized ambulatory video EEG recordings performed between March 2020 and September 2020 across 39 states through an in‐home diagnostic testing facility (Stratus). Details on procedure for in‐home monitoring have been detailed previously (see,^16^ Section 2.2). Anonymized database does not meet the definition of human subject research as personal identifiers have been removed from all reports. Thus, IRB approval for the current study was not required. Researchers followed STROBE guidelines for preparation of this manuscript.

### 2.2 Study population

**FIGURE 1:**
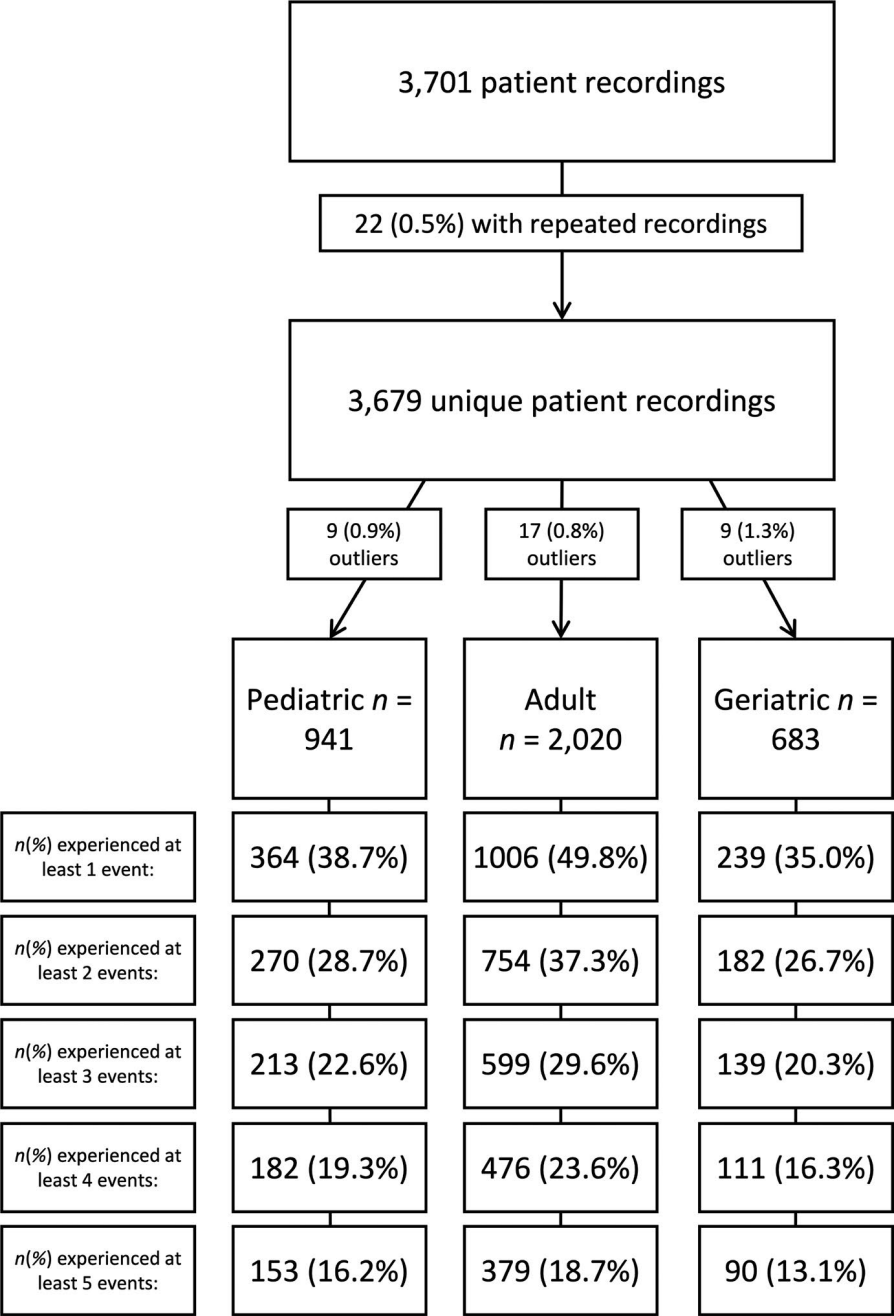
Diagram of retrospective review for in‐home patient recordings performed between March 2020 and September 2020. For this study, only completed recordings with available neurologist reports of EEG data were reviewed

Completed assessments for which neurologists' reads of EEG recordings were available were reviewed, resulting in a sample of 3701 patient recordings. For patients who performed multiple in‐home EEG assessments during this period (n = 22) only the first recording was included in analyses. Patients were divided into 3 cohorts based upon age (pediatric group below 18 years of age; adult group 18 years or older up to 65 years; and geriatric group 65 years and above). Group outliers were identified as +3 standard deviations (SD) above the group mean for number of push‐button events noted during recording (pediatric group n = 9, adult group n = 17, and geriatric group n = 9). This resulted in final sample size of 941 unique patients in the pediatric group, 2020 in the adult group, and 683 in the geriatric group. Figure 1 summarizes this process.

### 2.3 Variables

Final EEG reports were reviewed, and the number of push‐button events as well as the time of push‐button occurrences were recorded. Based upon patient self‐reported symptoms at the time of the events, each event was marked as either a typical or nontypical event, that is, whether the event corresponds to the symptom complaint that spurred the assessment. Additionally, all non‐push‐button seizure events, that is, neurophysiological events on the raw EEG record absent of any corresponding push‐button events, were noted and the times were recorded. Duration of time between EEG start and occurrence of either typical push‐button event or non‐push‐button seizure event was calculated and converted to hours.

**FIGURE 2:**
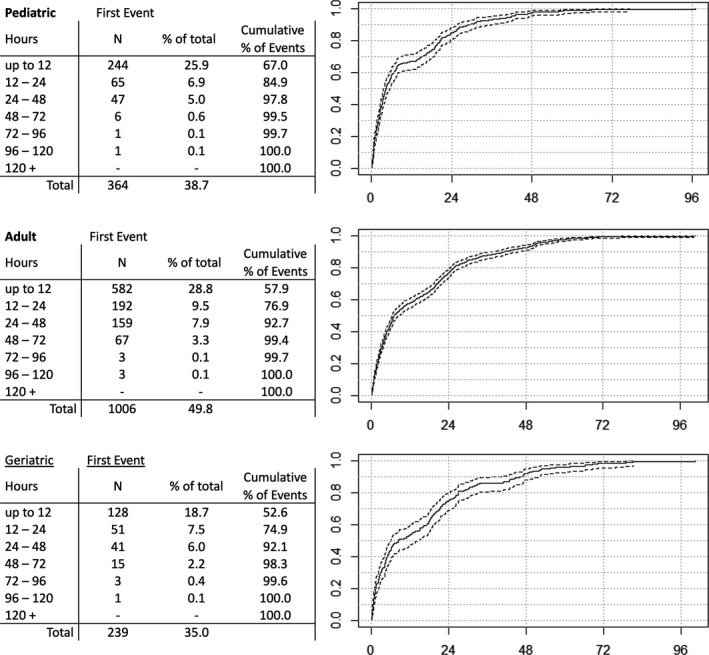
Frequency distributions of time to first event from start of EEG recording, grouped by age along with cumulative incidence curves

### 2.4 Statistical methods

Continuous variables such as participant age, total recording duration, and duration to the first, as well as mean number of, clinical events are characterized per cohort by calculating mean (*M*), standard deviation *(*SD*)*, minimum duration*(Min)*, maximum duration *(Min)*, and skew of distribution *(Skew)*. Median (*Md*) and interquartile range *(IQR)* are also calculated and reported for event times. Categorical EEG outcomes were characterized per cohort by frequency of occurrence (*%*). Within each cohort, a logistic regression was performed to determine the probability (odds ratio, *OR*) of observing a clinical event with each subsequent day of recording. The following exploratory analyses were included to accurately describe observed findings. The impact of EEG outcome on time to first event was also observed using Welch's ANOVA within each cohort. Additionally, cohorts were compared using Welch's ANOVA to determine differences in duration to first clinical event, as well as overall recording time, to accurately describe observed findings.

## 3 RESULTS

A total of 3644 unique outpatient studies were analyzed, including 941 studies from children, 2020 studies from adult patients, and 683 studies from geriatric patients. Demographic information for these patient cohorts and corresponding EEG characteristics are presented in Table 1.

**TABLE 1:** Demographic data and EEG characteristics grouped by age

	Pediatric (n = 941)		Adults (n = 2020)		Geriatric (n = 683)	
	N	*%*	N	*%*	N	*%*
*Sex*						
Male	504	53.6	764	37.8	322	47.1
Female	437	46.4	1256	62.2	361	52.9
*EEG result*						
Normal	499	53.0	1420	70.3	456	66.8
Abn‐Sz	41	4.4	54	2.7	12	1.8
Abn‐Ep	189	20.1	192	9.5	48	7.0
Abn‐Oth	212	22.5	354	17.5	167	24.5
*Push button*						
Event	434	46.1	1160	57.4	312	45.7
Typical events	359	38.2	993	49.2	230	33.7
Seizure events (no push‐button)	5	0.5	13	0.6	9	1.3
No Typ.	577	60.9	1014	50.2	447	65.4

	Pediatric (n = 941)			Adults (n = 2020)			Geriatric (n = 683)		
	M (SD)	Min/Max	Skew	M (SD)	Min/Max	Skew	M (SD)	Min/Max	Skew
Age (in years)	9.07 (5.42)	0.00/17.92	−0.04	40.48 (13.93)	18.00/64.92	0.04	74.64 (6.89)	65.00/94.67	0.69
Total Rec. duration	41.19 (20.03)	2.77/157.98	0.84	64.03 (20.08)	2.13/165.10	0.37	65.60 (21.30)	3.00/165.48	0.35
Number Typ.	5.68 (6.30)	1.00/39.00	2.48	5.36 (5.77)	1.00/36.00	2.28	4.96 (4.81)	1.00/27.00	2.18
Time to first Typ.	11.06 (14.18)	0.02/96.90	2.08	15.07 (16.95)	0.00/100.68	1.58	16.45 (18.79)	0.00/100.35	1.58
Time to second Typ.	15.92 (15.45)	0.02/100.85	1.52	21.73 (18.65)	0.00/143.35	1.37	24.13 (21.23)	0.00/114.62	1.22
Time to third Typ.	16.78 (15.41)	0.02/81.50	1.26	27.62 (20.40)	0.03/143.35	0.94	29.89 (23.52)	0.00/120.85	1.05
Time to fourth Typ.	18.92 (17.11)	0.13/130.08	2.17	30.66 (20.85)	0.03/143.35	0.93	32.39 (21.71)	0.00/112.57	0.95
Time to fifth Typ.	19.91 (15.62)	0.13/66.80	1.14	33.84 (21.38)	0.03/143.35	0.90	35.72 (22.67)	0.00/140.07	1.15

Abbreviations: Abn‐Ep, Abnormal EEG result with epileptiform activity (ie, spikes or sharp waves); Abn‐Oth, Abnormal EEG result with other abnormality (eg, diffuse slowing); Abn‐Sz, Abnormal EEG result with seizure episode; Typ., typical event.

### 3.1 Pediatric group

The average age of our pediatric group was 9.07 years (SD = 5.42, *Md* = 8.92, *IQR* = 4.17–14.17 years) (Table 1), and the average duration of in‐home EEG recording was <2 days (M = 41.19, SD = 20.03, *Md* = 42.87, *IQR* = 22.33–50.72 hours). Out of 941 pediatric recordings, there were 499 normal recordings (53.0% of sample), 41 recordings of seizure events (4.4%), 189 recordings of interictal epileptiform events (ie, spikes or sharp waves, 20.1%), and 212 recordings of other EEG abnormalities (eg, diffuse slowing; 22.5%). A one‐way ANOVA revealed that time to first typical event did not significantly differ between EEG outcome for the pediatric cohort, *Welch's* F(3,124.875) = 0.799, *P* = .497. Overall, 434 recordings (46.1%) had push‐button events noted, with only 359 recordings (38.2%) experiencing at least one typical event. Five recordings (0.5%) had at least one seizure event with no push‐button events noted.

Three hundred sixty‐four pediatric patients experienced either typical nonepileptic (n = 331) or seizure events (n = 33) as their first recorded event. Of these patients, the average time to first event was 11.06 hours (SD = 14.18, *Md* = 4.27., *IQR* = 1.55–18.67). Frequency distributions (Figure 2) reveal that although a majority of patients experience their first events within 12 hours (n = 244, 25.9% of pediatric sample, 67.0% of first events), there is a relative yield increase associated with extended recording durations. A relative increase of 17.9% (n = 65) was observed extending to 24 hours and 12.9% (n = 47) observed to 48 hours. The relative yield is much lower after 48 hours, with 1.7% of first events occurring between 48 and 72 hours, and 0.5% after 72 hours. Potential confounding factors impacting this steep decline in relative yield after 48 hours are discussed further in the limitations section below.

**TABLE 2:** Frequency distributions of time to second, third, and fifth (mean) event from start of EEG recording, grouped by age

Hours	Second event			Third event			…	Fifth event		
	N	% of total	Cumulative % of events	N	% of total	Cumulative % of events		N	% of total	Cumulative % of events
**Pediatric**										
Up to 12	137	14.6	50.7	104	11.1	48.8		55	5.8	35.9
12–24	68	7.2	75.9	51	5.4	72.8		53	5.6	70.6
24–48	56	6.0	96.7	50	5.3	96.2		34	3.6	92.8
48–72	8	0.9	99.6	7	0.7	99.5		11	1.2	100.0
72–96	‐	‐	99.6	1	0.1	100.0		‐	‐	100.0
96–120	1	0.1	100.0	‐	‐	100.0		‐	‐	100.0
120 +	‐	‐	100.0	‐	‐	100.0		‐	‐	100.0
Total	270	28.7		213	22.6			153	16.3	
**Adult**										
Up to 12	290	14.4	38.5	157	7.8	26.2		58	2.9	15.3
12–24	197	9.8	64.6	148	7.3	50.9		82	4.1	36.9
24–48	188	9.3	89.5	185	9.2	81.8		147	7.3	75.7
48–72	73	3.6	99.2	95	4.7	97.7		78	3.9	96.3
72–96	3	0.1	99.6	12	0.6	99.7		11	0.5	99.2
96–120	2	0.1	99.9	1	0.0	99.8		2	0.1	99.7
120 +	1	0.0	100.0	1	0.0	100.0		1	0.0	100.0
Total	754	37.3		599	29.6			379	18.7	
**Geriatric**										
Up to 12	64	9.4	35.2	36	5.3	25.9		12	1.8	13.3
12–24	46	6.7	60.4	27	4.0	45.3		17	2.5	32.2
24–48	46	6.7	85.7	48	7.0	79.9		37	5.4	73.3
48–72	23	3.4	98.4	20	2.9	94.2		20	2.9	95.6
72–96	1	0.1	98.9	6	0.9	98.6		3	0.4	98.9
96–120	2	0.3	100.0	1	0.1	99.3		‐	‐	98.9
120 +	‐	‐	100.0	1	0.1	100.0		1	0.1	100.0
Total	182	26.7		139	20.3			90	13.1	

For patients that reported typical clinical events during recording, an average of 5.68 clinical events (SD = 6.30, *Md* = 4.0, *IQR* = 2.0–7.0 events) were noted. The average time to the mean number of subsequent events is presented in Table 1. For children, the average duration to the first five typical events occurred within 24 hours (mean time to 5th typical event = 19.91, SD = 15.62, *Md* = 18.65, *IQR* = 6.48–26.15, n = 153, 16.3% of pediatric sample), with 100% of those patients capturing the fifth clinical event prior to 72 hours (Table 2).

In looking at patients who completed their recording without any typical clinical events and no notable EEG abnormality, 317 patient recordings (33.7%) were identified. Out of those recordings, 117 (36.9% relative) were recordings that were <24 hours, 116 (36.6% relative) terminating between 24 and 48 hours, 76 (24.0% relative) terminating between 48 and 72 hours, and a final 8 (2.5% relative) terminating between 72 and 96 hours. A logistic regression was performed to determine how additional recording days predicted the likelihood of observing a clinical event in the pediatric sample. Age and medication status (whether the person was on antiseizure medication) were also included in the model as potential confounding factors. The overall model was significant, *X* ^2^ (3) = 27.207, *P* < .001, accounting for around 4% of variance observed*, Nagelkerke R* ^2^ *= *0.039. As age increased, the likelihood of observing an event slightly decreased around 4%, *Wald's statistic* = 7.004, *P* = .008, *OR* = 0.967, *95% CI* [.943,0.991]. Alternately, each additional day of recording increased the likelihood of observing an event by about 40%, *Wald's statistic* = 19.259, *P* *< *.001, *OR* = 1.410, *95% CI* [1.209,1.643]. Medication status did not significantly impact the likelihood of observing events, *Wald's statistic* = 3.102, *P = *.078, *OR* *= 1*.273, *95% CI* [.973,1.664].

### 3.2 Adult group

The average age of our adult group was 40.48 years (SD = 13.93, *Md* = 40.17, *IQR* = 28.04–52.81 years), and average duration of recording was <3 days (M = 64.03, SD = 20.08, *Md* = 66.38, *IQR* = 59.31–69.51 hours). Out of a total of 2020 recordings, there were 1420 normal recordings (70.3% of sample), 54 recordings of seizure events (2.7%), 192 recordings of interictal epileptiform events (ie, spikes or sharp waves, 9.5%), and 354 recordings of other EEG abnormalities (17.5%). A one‐way ANOVA revealed that time to first typical event did not significantly differ between EEG outcome for the adult cohort, F(3,1005) = 0.996, *P* = .394. In the adult sample, 1160 recordings (57.4%) had at least one push‐button event recorded and 993 recordings (49.2%) with at least one typical event. Thirteen recordings (0.6%) had at least one seizure event noted on the report with no push‐button events recorded.

One thousand fourteen adult patients experienced either typical nonepileptic (n = 967) or seizure events (n = 39) as their first recorded event. The average time to first event for these patients was 15.07 hours (SD = 16.95, *Md* = 7.03, *IQR* = 2.36–22.92 hours). The frequency distribution of events across time (Figure 2) reveal that just over half of patients experienced their first events within 12 hours (n = 582, 28.8% of adult sample, 57.9% relative). As with the pediatric cohort, there was an incremental yield observed, with a relative increase of 19.0% (n = 192) to 24 hours, 15.8% (n = 159) to 48 hours, and 6.7% (n = 67) to 72 hours. An additional 6 patients (0.2% of adult sample, 0.6% relative) experienced their first event beyond 72 hours.

Patients reporting typical push‐button events averaged 5.36 typical events during recording (SD = 5.77, *Md* = 3.0, *IQR* = 2.0–7.0 events). The average time to subsequent events is presented in Table 1. For the adult sample, the average duration to the first five typical events occurred within 48 hours (mean time to 5th typical event = 33.84, SD = 21.38, *Md* = 28.45, *IQR* = 19.87–47.67 n = 379, 18.8% of adult sample), and 96.3% of those experiencing at least 5 typical events noted their fifth event prior to 72 hours. An additional 13 patients (3.4%) noted their fifth typical events occurring between 72 and 120 hours, and 1 patient reported their fifth typical event occurring after 120 hours of recording (Table 2).

In looking at patients who completed their assessment without any typical clinical events or any notable EEG abnormality, 708 patient recordings (35.0%) were identified. Out of those recordings, 52 (7.3% relative) were recordings that were <24 hours, 109 (15.4%) terminated between 24 and 48 hours, and the majority, 464 (65.5%) terminated between 48 and 72 hours. An additional 50 (7.1%) terminated between 72 and 96 hours and 33 (4.6%) terminated after 96 hours. A logistic regression was performed to determine how additional recording days predicted the likelihood of observing a clinical event in the adult sample. Age and medication status were also included in the model as potential confounding factors. The overall model was significant, *X* ^2^ (3) = 20.836, *P* < .001, accounting for around 1% of variance observed, *Nagelkerke R^2^* = 0.014. For each additional day of recording, the likelihood of observing an event increased by about 25%, *Wald's statistic* = 16.865, *P* < .001, *OR* = 1.247, *95% CI* [1.122, 1.385]. Patient age, *Wald's statistic* *= 0*.349, *P = *.555, *OR* = 0.998, *95% CI* [0.992, 1.004], and medication status, *Wald's statistic* = 3.423, *P* = .064, *OR* = 1.207, *95% CI* [0.989, 1.472], did not significantly impact the likelihood of observing events within this cohort.

### 3.3 Geriatric group

The mean age of our geriatric group was 74.64 years (SD = 6.89, *Md* = 73.50, *IQR* = 68.92–78.75 years), and average duration of recording was <3 days (M = 65.60, SD = 21.30, *Md* = 67.18, IQR = 62.65–69.93 hours). Of a total of 683 geriatric studies, there were 456 normal recordings (66.8% of sample), 12 recordings of seizure events (1.8%), 48 recordings of epileptiform events (ie, spikes or sharp waves, 7.0%), and 167 recordings of other EEG abnormalities (24.5%). A one‐way ANOVA revealed that time to first typical event significantly differed between EEG outcome for the geriatric cohort, *Welch's* F(3,48.726) = 7.241, *P* < .001, with Games‐Howell post hoc analyses revealing that those with a diagnosis of seizure events experienced first clinical event significantly sooner than both those with normal EEGs, mean difference = −9.49, *P* < .001, and with other EEG abnormalities, mean difference = −9.38, *P* = .010. In the geriatric sample, 312 recordings (45.7%) had at least one push‐button event recorded, with 230 recordings (33.7%) with at least one typical event. Nine recordings (1.3%) had at least one seizure event noted on the report with no push‐button events recorded.

Two hundred thirty‐nine geriatric patients experienced either typical nonepileptic (n = 229) or seizure events (n = 10) as their first recorded event. Of these, the average time to first event was 16.45 hours (SD = 18.79, *Md* = 8.40, *IQR* = 2.17–24.08). Frequency distributions (Figure 2) reveal that approximately half of patients (n = 128, 52.6% relative) experienced their first event within the first 12 hours. The incremental yield for additional time was 22.3% (n = 51) in 24 hours, 17.2% (n = 41) in 48 hours, and 6.2% (n* = *15) in 72 hours. Four additional patients (0.5% of geriatric sample, 1.7% relative) experienced their first events between 72 and 120 hours.

Patients reporting typical push‐button events averaged 4.96 typical events during recording (SD = 4.81, *Md* = 3.0, *IQR* = 2.0–7.0 events). The average time to subsequent events is presented in Table 1. For the geriatric sample, the average duration to the first five typical events occurred within 48 hours (mean time to 5th typical event = 35.57, SD = 22.67, *Md* = 32.40, *IQR* = 21.22–48.26, n = 90, 13.2% of geriatric sample), and 95.6% of patients reporting at least five events noted the fifth event occurred prior to 72 hours recording. An additional 3 patients (3.3%) noted their fifth events occurring between 72 and 96 hours and one patient noted a fifth event after 120 hours (Table 2).

In looking at patients who completed their recording without any typical clinical events and no notable EEG abnormality, 305 patient recordings (44.7%) were identified. Out of those recordings, 25 (8.2% relative) were recordings that were <24 hours, 32 (10.5%) terminated between 24 and 48 hours, and the majority, 206 (67.5%) terminated between 48 and 72 hours. An additional 28 (9.2%) terminated between 72 and 96 hours and 14 (4.6%) terminated after 96 hours. A logistic regression was performed to determine how additional recording days predicted the likelihood of observing a clinical event in the geriatric sample. Age and medication status were also included in the model as potential confounding factors. The overall model was significant, *X* ^2^ (3) = 15.605, *P* = .001, accounting for around 3% of variance observed, *Nagelkerke R^2^ = *0.031. For the geriatric cohort, increased age resulted in a lower likelihood of capturing an event of around 3%, *Wald's statistic* = 5.888, *P* = .015, *OR* = 0.971, *95% CI* [0.948, 0.994]. For each additional day of recording, the likelihood of observing an event increased by about 25%, *Wald's statistic* = 5.746, *P* = .017, *OR* = 1.248*95% CI* [1.041, 1.497]. Age and recording duration were significant at *P* < .05; however, due to the number of comparisons performed, these should be interpreted with caution. Medication status did not significantly impact the likelihood of observing events, *Wald's statistic* = 1.549, *P* = .213, *OR = *0.804, *95% CI* [0.571, 1.133].

### 3.4 Comparisons among groups

To characterize the frequency of repeated ambulatory EEG use, all cohorts were reviewed for repeated testing within 1 year prior and 1 year posttesting, revealing small percentages within each cohort (pediatric prior: n = 21, 2.23%/post: n = 34, 3.61%; adult prior: n = 26, 1.29%/post: n = 15, 0.74%; geriatric prior: n = 8, 1.17%/post: n = 3, 0.44%).

Exploratory analyses were performed to assess whether the time to first event differed by group. As time to first event is not normally distributed, a log transformation was performed and used for the following analyses. A one‐way ANOVA revealed that time to first typical event significantly differed among the age groups, *Welch's* F(2,529.851) = 9.529, *P* < .001. Games‐Howell post hoc analyses revealed that children on average had first events occur significantly sooner compared to the adult cohort, mean difference = −0.42, *P* < .001, and the geriatric group, mean difference = −0.39, *P* = .016. Alternately, the adult and geriatric groups did not significantly differ, mean difference adult‐geriatric = 0.03, *P* = .963. Means for groups in hours listed in Table 1, revealing that children, on average, had events occur around 4 hours sooner than adult patients and over 5 hours sooner than geriatric patients.

Similarly, a one‐way ANOVA revealed that total duration of recording significantly differed among age groups, *Welch's* F(2,1588.093) = 458.605, *P* < .001. Games‐Howell post hoc analyses indicated that although the adult and geriatric groups did not differ significantly on recording time, mean difference of adult‐elderly = −1.57 hours, *P* = .210, children were recorded for a significantly shorter amount of time compared to either the adult, mean difference = −22.84 hours, *P* < .001, or the geriatric group, mean difference = −24.41 hours, *P* < .001.

## 4 DISCUSSION

In‐home EEG has demonstrated equitable diagnostic outcomes compared to gold standard inpatient monitoring^16^ at a significantly lower cost,^10^ making diagnostic assessments more accessible to a wider range of patient populations. However, in‐home assessments are only effective if used appropriately. Effective test usage not only includes prescribing the appropriate test for each patient,^17^ but also optimizing the likelihood of observing clinically important events by ordering examinations of sufficient duration to capture the necessary information.

For children, the data indicate that this cohort experienced the first typical event earlier than adult or geriatric patients. The average time to first typical event in children was <12 hours into the study. However, in practice, this may not be instructive, as 43.4% of the events observed would not have been captured within this time frame. The current analyses reveal that likelihood of capturing an event increased by 40% for each additional day recorded, yet the frequency distributions highlight that the incremental yield seems to drop after 48 hours of recording. This may be a function of physician ordering habits as children were recorded for significantly shorter durations compared to either adult or geriatric groups. The data show that over two‐thirds of nondiagnostic studies (ie, those that did not record any clinical events) were <48 hours, which may significantly limit the ability to observe any potential events beyond this time frame. Based upon the current data, a recording duration of at least 48 hours for children seems adequate to maximize the likelihood of recording at least one typical event.

Results for the adult and geriatric cohorts do not differ significantly from each other. Of the first typical events captured, approximately 75% of those were recorded within the first 24 hours. These findings represent a higher frequency than previously reported for an adult population,^18^ but lower than estimates for a geriatric population.^15^ In our data, the incremental yield increased by 15%–17% for adult and geriatric groups, respectively, by 48 hours, and an additional 6% for both groups by 72 hours, capturing up to 98% of all first events by the third day. Similar to the pediatric sample, we see a potential ceiling effect in that the majority of nondiagnostic studies discontinue around 72 hours. This may limit potential observations beyond this time point. Based upon the findings presented, though, EEG recording duration of at least 72 hours should be sufficient to maximize likelihood of recording at least one typical event.

Thus far, the field has not been successful to identify clinical determinants that predict seizure capture during a recording, for example, seizure frequency prior to assessment,^3^ and the heterogeneity of seizure disorders may limit the standardization of video EEG recording guidelines across all types of patients. In the current data 19.3% (n = 182) of the pediatric cohort, 35.2% (n = 712) of the adult cohort, and 22.1% (n = 151) of the geriatric cohort successfully recorded the typical events and were ruled normal EEG. However, 33.7% (n = 317) of the pediatric sample, 35.0% (n = 708) of the adult sample, and 44.7% (n = 305) of the geriatric sample resulted in recordings with no observable abnormalities nor typical events. Although these numbers are consistent with previous retrospective research, for example, 30%,^13^ 33%,^8^ and 47%,^19^ a recent report on nonvideo ambulatory EEG revealed lower yields (37.7% of positive studies, or 62.3% of nondiagnostic results).^20^ Critically, the authors note that average duration for these recordings was shorter (37.7 hours; 21) than typically noted in the literature, or the present study (average duration for adult and geriatric in the current study is >64 hours). Within our data, nondiagnostic normal findings are almost double for the pediatric and geriatric populations relative to diagnostic normal findings. As this is a retrospective study, we cannot know whether these patients would have experienced a typical event if given unlimited recording time.

A pivotal factor to consider is the reason for the assessment and letting this guide timelines for recordings accordingly. The optimal times offered above are for detection of single clinical event; however, if the goal of the assessment is to characterize seizure occurrence or location, multiple events may need to be captured, which would necessitate longer recordings. Additionally, for the adult and geriatric cohorts, there were individual patients who benefited from recordings beyond 72 hours, a finding that is in line with previous work suggesting that, as long as the patient is amenable, there is no upper limit for EEG recordings in EMUs beyond which further recording is futile.^21^

There are several limitations to the current study as retrospective analyses limit the research questions that can be addressed. The current study utilizes a large and diverse sample to characterize timing of events for three distinct age groups. However, the current data could represent a self‐selected sample of the population, as particular patients may be more amenable to, or appropriate for, in‐home EEG and therefore results would not generalize to every patient. Importantly, the frequency of seizure‐like events prior to assessment was not systematically reported in the database, and therefore, the current study cannot determine the impact this has on the duration to first event. Individual physician ordering habits may also inadvertently bias the sample. Unfortunately, the retrospective review does not allow us to know the primary or suspected indication supporting the assessment. It is possible that some patients in this sample performed an in‐home assessment for a suspected diagnosis other than epilepsy or nonepileptic seizures, for example, sleep disturbances or behavioral changes following head trauma. For these patients, the goal may not be detection of a seizure‐like event, and therefore, the duration is not informed by this metric. Although this may introduce noise into our logistic regression, we still observed a clear linear relationship between recording duration and potential for observing a typical clinical event. Alternately, the length of the recording itself may impact our results. As discussed above, an observable ceiling effect occurs in each of the subsamples, with approximately 30%–45% of the sample discontinuing services without EEG abnormality or experiencing a typical event, which could reflect either a unique subsample of the population that is prone to null EEG findings or a conservative use of in‐home EEG. Future prospective work should examine whether there are characteristics unique to those that fail to observe clinically relevant information during typical recording durations, and whether this subset of individuals would uniquely benefit from extended studies beyond 48 hours in children and 72 hours in adults/geriatrics.

The field may also benefit from additional work assessing the use of repeat testing in both ambulatory and EMU settings, particularly the diagnostic yield of ambulatory EEGs after nondiagnostic EMU admissions. The current dataset demonstrated a relatively small percentage of repeated assessment within a year of assessment (<4% for children, <1.5% for adult/geriatric cohorts). There are limited conclusions that can be drawn from the current data; however, an important question for future research would be to assess whether repeated testing is consistently low across monitoring settings, and if not, the reasons for repeated testing and the impact on medical costs to both the patient and the medical industry.

In‐home EEG monitoring may be a viable and important alternative to inpatient monitoring for patients across a wide range of age cohorts when used effectively. Although future prospective work is needed to validate the current observation, the results presented suggest that an optimal EEG recording duration to observe at least one typical event is at least 48 hours for children and between 48 and 72 hours for adult and geriatric patients to maximize likelihood of event capture to facilitate clinical diagnosis and management.

## CONFLICT OF INTEREST

HK and JS are employees of Stratus, a neurodiagnostic company specializing in in‐home EEG monitoring. Stratus is also the funder for the current research program. No other authors report any conflicts of interests or disclosures relating to this manuscript. We confirm that we have read the Journal's position on issues involved in ethical publication and affirm that this report is consistent with those guidelines.
